# Continued dysregulation of the B cell lineage promotes multiple sclerosis activity despite disease modifying therapies

**DOI:** 10.12688/f1000research.74506.2

**Published:** 2022-08-08

**Authors:** Ana C. Londoño, Carlos A. Mora

**Affiliations:** 1 Neurologia y Neuroimagen, Instituto Neurologico de Colombia (INDEC), Medellin, Antioquia, Colombia; 2 Spine & Brain Institute, Ascension St. Vincent's Riverside Hospital, Jacksonville, FL, 32204, USA

**Keywords:** Multiple sclerosis, antibody secreting cell, memory B cell, naïve B cell, B regulatory cell

## Abstract

A clear understanding of the origin and role of the different subtypes of the B cell lineage involved in the activity or remission of multiple sclerosis (MS) is important for the treatment and follow-up of patients living with this disease. B cells, however, are dynamic and can play an anti-inflammatory or pro-inflammatory role, depending on their milieu. Depletion of B cells has been effective in controlling the progression of MS, but it can have adverse side effects. A better understanding of the role of the B cell subtypes, through the use of surface biomarkers of cellular activity with special attention to the function of memory and regulatory B cells (Bregs), will be necessary in order to offer specific treatments without inducing undesirable effects.

## Introduction

Multiple sclerosis (MS) is a chronic, neuroinflammatory disease of autoimmune origin, which causes demyelination and neurodegeneration of the central nervous system (CNS). It is the leading cause of disability among young adults with neurological diseases. ^1^ Current MS diagnosis methodologies are based on criteria that include clinical presentation, determination of oligoclonal bands (OCB) and other biomarkers in the cerebrospinal fluid (CSF), as well as presence of inflammatory and/or demyelinating lesions in the magnetic resonance imaging (MRI) analysis. Currently, the follow-up and response to treatment of patients with MS is based on the concept of “no evidence of disease activity” (NEDA) based on clinical presentation, imaging, and disability progression, without taking into account any other biomarkers of disease. Over the last three decades, the prognosis of disease has dramatically improved due to the availability of multiple disease modifying therapies (DMT).

It has been established that both T and B cells play a role in the pathogenesis of MS. ^2, 3^ The MS-promoting role of B cells can be carried out through the secretion of antibodies such as OCB, presentation of antigens, activation of T cells and/or production of cytokines. ^4^ Perturbation of T cell homeostasis due to a reduction in cells of thymic origin, and reduction in the diversity of T cell repertoires, among others, has been documented in MS. ^5^ Antibody secretion is the most studied function in the pathogenesis of MS and, in recent years, exploration of the role of cytokines in the regulation of immunity, for therapeutic purposes, has begun. In addition, the use of anti-CD20 therapies for MS, which do not affect plasmablasts (PB) and plasma cells (PC), has led to a better understanding of the role of B cells in the pathogenesis of the disease. ^4^ Although DMT have the ability to slow down the progression of the disease, they are not a cure. In the present article, we show how the dysregulation of the B cell lineage could be strongly linked to the activity and progression of the disease and how selective therapy, guided by cell surface markers, could become key in controlling it.

### B cell lineage

**Figure 1.**
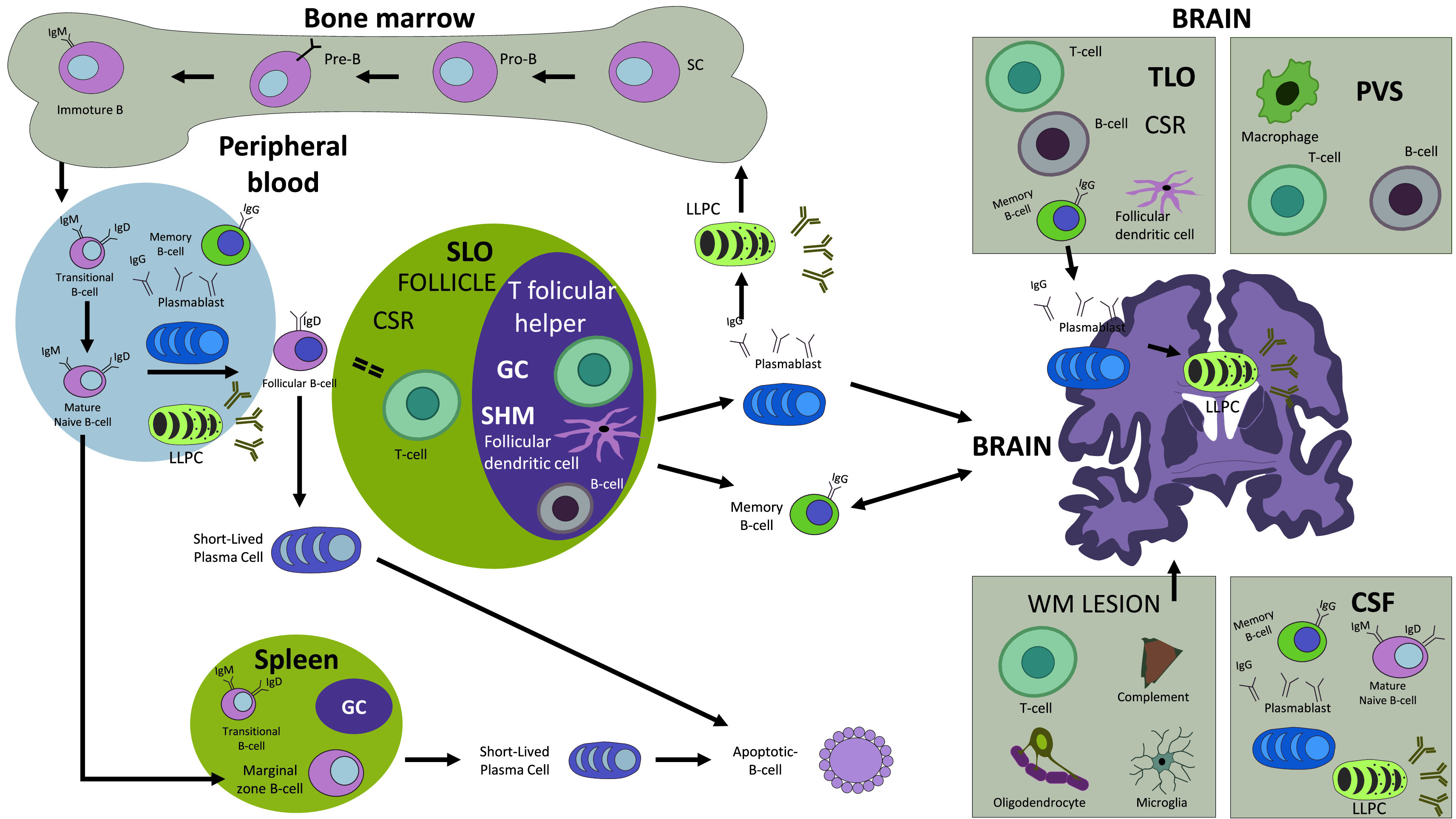
The B cell lineage cycle. The B cell lineage begins in fetal life from pluripotent hematopoietic stem cells (SC) in the fetal liver and in the postnatal bone marrow developing B cell receptors and migrating to different locations, including peripheral blood, and the secondary lymphoid organs (SLO) where they will acquire, in the germinal center (GC), the ability to recognize antigens and produce highly specific antibodies. In the pathogenesis of multiple sclerosis (MS), these cells may cross the BBB and may be found in the CSF, perivascular spaces (PVS), white matter (WM) demyelinating lesions and in the tertiary lymphoid organs (TLO). Abbreviations: SC: stem cell; SLO: secondary lymphoid organ; CSR: class switch recombination; SHM: somatic hypermutation; GC: germinal center; TLO: tertiary lymphoid organ; PVS: perivascular space; WM: white matter.

The family of B cell subsets results from an evolutionary process of embryonic cells expressing different surface markers (especially CD19, CD20, and CD38) through their lifespan, in different organs, until they reach the state of antibody secreting cells (ASC), thus culminating the evolution process with the presence of effector B cells. ^6^ The change of stage from membrane-linked antibody cell to ASC represents the terminal differentiation toward B cells that do not proliferate ^7, 8^ ( Figure 1). The B cell lineage begins in fetal life from pluripotent hematopoietic stem cells (SC), located in the fetal liver and in the postnatal bone marrow (BM). Henceforth, they evolve into multipotent myeloid/lymphoid progenitors (MPP), which continue their evolution towards the common lymphoid progenitors (CLP). ^6^ CLP from the BM evolve to a pro-B state in which they express the CD19 marker and then transform into pre-B cells; those, in addition to expressing CD19, begin to express CD20, and later progress to immature B cells that express IgM as a surface marker. ^6^ While transiting from the BM to the secondary lymphoid organs (SLO), the B cells express the B cell receptor (BCR) surface markers IgM and IgD, thus evolving into transitional B cells; this step requires a checkpoint that entails clonal deletion and receptor editing before entering the SLO (spleen, lymphoid node, tonsils, and mucosa-associated lymphoid tissue [MALT]) where they become mature naïve B cells. ^6^ The mature naïve B cells, at this point, can have three possible destinations: a) they enter the marginal zone of the spleen where they may become short-lived plasma cells (SLPC) that produce IgM and rapidly enter apoptosis (since these are B cells involved in rapid and transitory defense); b) move to the intestine and the pulmonary epithelium (B1 cells); c) migrate to splenic follicles and lymphoid nodules, becoming follicular B cells. ^9, 10^ Naïve B cells that carry the BCR IgD go through early class switch recombination (CSR) in the extrafollicular zone, with support from T cells, then enter the germinal center (GC) where they undergo somatic hypermutation (SHM), after which they express BCR IgG. ^11^ Subsequently, the resulting memory B cells, PB and PC will have the ability to secret high-affinity antibodies for decades, or for the lifespan of an individual, and most of them will be able to migrate to the bone marrow to establish as long-lived plasma cells (LLPC). ^11– 13^ Dysregulation of the GC has been associated with autoimmune disease. ^14^ Evidence suggests that the origin of B cell autoreactivity occurs in the GC due to dysfunction of thymus-derived follicular T helper cells and follicular regulatory T cells. ^15^ PB may develop from any type of activated B cell (including naïve, marginal zone, follicular, and memory B cells), but it is not clear if PB that originated from these cells (except for memory B cells) are competent to mature to LLPC. ^6^ PB will carry the CD19 ^+^CD20 ^-^CD27 ^++^CD38 ^++^IgG ^+/-^ markers, and will express the chemokine receptor CXCR4, which will help them get attracted to the chemokine CXCL12 in the BM niches. As an alternative, PB expressing the receptor CXCR3 will become LLPC in the spleen and lymph nodes (assisted by the chemokine CXCL12) or in inflamed tissue (assisted by the chemokines CXCL9-CXCL12), and subsequently undergo apoptosis upon resolution of inflammation. ^7^ Hereafter, memory B cells access the CNS through the disrupted blood brain barrier (BBB). They are identified in perivascular spaces, demyelinating lesions in the brain and spinal cord, and disperse in the meninges where they can form aggregates known as tertiary lymphoid organs (TLO). These TLO emulate GC function, supporting the formation and persistence of cortical lesions. ^16– 19^ In addition, they are a local source of class-switch IgG that contribute to the immune process and are subsequently determined as OCB in the CSF of patients with MS. ^20^ In the meninges, the inflammatory infiltrates are composed of CD3 ^+^ T cells, CD20 ^+^ B lymphocytes and PC. ^16^ In the white matter lesions, the inflammatory infiltrates are localized in the perivascular spaces containing T and B lymphocytes and PC. ^16^ In the diffuse infiltrates and normal-appearing white matter, CD8 ^+^ T lymphocytes predominate almost exclusively. ^16^ The presence of PB in CSF has been reported. ^21, 22^ Despite the knowledge accumulated to this date, the complete understanding of the evolution of the B cell lineage is still in progress. Activated lymphocytes are able to access the CNS, both in health and disease, through the BBB, the blood meningeal barrier, and the blood-CSF barrier. ^12^ In normal conditions the amount of B cells that access the CNS is very low. ^23^ These cells primarily exit the CNS via lymphatic drainage through nasal blood vessels, or via meningeal lymphoid vessels to the lymphoid cervical nodules. ^24^

Naïve and memory B cells are crucial within the B cell lineage and they have been shown to negatively correlate in their function: increased memory B cells and decreased naïve B cells have been linked with a worsening of the disease, and vice-versa. ^5^ In fact, when the presence of memory B cells induce the auto-proliferation of CD4 T cells, which tend to home in the brain, naïve B cells are decreased. ^5^ Memory B cells can be heterogeneous, i.e., originate from different cells or express different phenotypes, including class-switched (CD19 ^+^CD27 ^+^IgM ^-^IgD ^-^) and class-unswitched (CD19 ^+^CD27 ^+^IgM ^+^IgD ^-^). ^25^ Inhibition of memory B cells prevents relapsing MS. ^25^ In a study in naïve patients with relapsing remitting MS (RRMS), the interaction between T and B cells was documented, highlighting that B cells act as antigen presenting cells (APC) to auto proliferating CD4 ^+^ T cells. ^5^ Increased amounts of T cell co-stimulatory proteins and major histocompatibility complex (MHC) class II molecules are expressed by B cells in the periphery (blood and SLO), and in the CSF and CNS of patients with MS. ^12^ Patients in remission carrying the HLA-DR15 ^+^ marker showed an increase in auto-proliferative B and T cells and a decrease in naïve B cells. ^5^ On the other hand, treatment with anti-CD20 was associated with a decrease in auto-proliferative memory T and B cells and increased presence of naïve B cells. ^5^

### Regulatory B cells

Within the B cell lineage, the regulatory B cells subset (Bregs) stands out, since there is still no agreement on its origin and classification. Bregs are not a specific subtype of B cells, but represent a regulatory functional state resulting from inflammation. ^26^ Although it has been assumed that interleukin 10 (IL10) is the hallmark of Bregs, other factors such as IL35, transforming growth factor (TGF) β, and direct cell-to-cell contact are also mechanisms of Bregs function. ^26^ Immature B cells, mature B cells, PB and PC are believed to function as Bregs. ^26^ The Bregs can express the following markers: IL10, CD27, CD5, CD25, CD86, CD24 and CD28. ^27^ Based on the production of IL10, three important subtypes of regulatory Bregs have been identified, including the transitional (CD19 ^+^CD24 ^high^CD38 ^high^), naïve (CD19 ^+^CD24 ^+^CD38 ^+^), and memory (CD19 ^+^CD24 ^high^CD38 ^-^) subtypes, among which the transitional cells are the main producers of IL10. ^28^ Transitional B cells are capable of suppressing differentiation of naïve T cells into Th1 and Th17, which are dependent on the co-stimulatory molecules CD80 and CD86. ^29^ Survival of Bregs is linked to the B cell activating factor (BAFF) and to a proliferation inducing ligand (APRIL). ^20^ Under normal conditions, the population of Bregs is kept low in order to maintain immune homeostasis. ^26^ In newborns, 50% of umbilical cord blood B cells correspond to the transitional B cell subtype whereas, in adults, it represents only 4% of the cell population in peripheral blood. ^28^ It is estimated that, in human peripheral blood, the Bregs subtype represents only 1-2% of all B cells. ^29^ In an experimental allergic encephalitis (EAE) animal model, it was documented that IL10 contributed to the reduction of the inflammatory response mediated by microglia and astrocytes in the CNS. ^30^ Bregs transfer reversed the increase in Th1 and Th17 cells in an arthritis model lacking IL10. ^31^ In mice with low expression of the IL35 subunit p35, or EBi3 in B cells, an inability to recover from EAE was detected, with evidence of activation of macrophages and inflammatory T cells, and an increased activity of B cells such as APC. ^32^ IL10 regulates the differentiation of the lineage from IL10-secreting B cells to PB that secrete IgG or IgM. ^33^ In a post-mortem analysis of MS patients with high levels of meningeal inflammation and cortical demyelination, Magliozzi *et al*., reported an increase in IL10 expression, among other proinflammatory cytokines and molecules related to B cell activity and lymphogenesis in meninges and CSF. Additionally, an increase in IL10 was found in the CSF of MS patients with high cortical involvement at the time of diagnosis. ^34^ Early development of MS in individuals with the clinically isolated syndrome (CIS), or radiologically isolated syndrome (RIS), seems to correlate with a reduced production of IL10 by B cells. ^35^ In a cohort of MS patients followed for 10 years, Farian *et al.* found that patients with positive OCB at diagnosis advanced, more frequently and earlier in the course of the disease, to a progressive phase. ^36^ This can be explained by the presence of a greater intrathecal inflammatory component that causes greater cortical involvement. ^36^ Besides, the authors reported an over-expression of inflammatory molecules, including IL10. ^36^ PB and PC inside the MS lesions presented a high IL10 expression. ^37^

### Double negative and CD21 ^LOW^ cells

Another subset of CD19 ^+^ B cells that could be relevant to MS pathogenesis are the double negative (DN) B cells (IgD ^-^CD27 ^-^) and the CD21 ^low^ cells, which have been associated with aging and autoreactivity. ^38^ These cells are believed to develop outside the GC, are independent from T-cells, and display a pro-inflammatory cytokine profile. ^38^ These cells have been found in healthy subjects, and have also been found at higher levels in the CSF of MS patients younger than 60 years when they were compared to age-matched healthy donors (DN B cells 19.5% against 3.03%, and CD21 ^low^ 21.95% against 6.06%, respectively). ^38^ Most DN B cells display an IgG ^+^ phenotype while CD21 ^low^ B cells originate from a heterogeneous population that includes CD27 ^-^ naïve, CD27 ^+^ memory, and IgG ^+^ and IgM ^+^ B cells. ^38^ Both DN and CD21 ^low^ B cell frequencies were higher in the CSF compared to blood levels for these patients. ^38^ Fraussen *et al*. have suggested that the DN B cells may have multiple origins, considering IgG ^+^ cells better linked to the class-switched memory B cells, while IgM ^+^ cells share more similarity with the naïve and the non-class-switched IgD ^+^CD27 ^+^ memory B cells. ^39^

### B cells in the CSF compartment in MS

Inflammation of the CNS is reflected in the presence of B cells in the CSF. ^21^ Cepok *et al.* evaluated the B cell subtype in CSF in MS patients, finding that the majority of detectable cells were memory B cells (CD19 ^+^CD27 ^+^), whereas a minority were naïve B cells (CD19 ^+^CD27 ^-^); those were different from naïve B cells that predominated in peripheral blood. ^21^ In addition, they detected PB (CD19 ^+^ CD27 ^++^CD138 ^+^CD38 ^+^) subtypes representing between 30-50% of cells in CSF, and were present in the course of the disease, without correlation with the level of PB present in peripheral blood, while short lived PB (CD19 ^+^CD27 ^++^CD138 ^+^CD38 ^++^HLADR ^++^) and PC (CD19 ^+/-^ CD27 ^++^CD138 ^+^CD38 ^+^HLADR ^-^) were absent from CSF. ^21^ In contrast, Corcione *et al*. reported the predominance of both memory B cells and PC in the CSF of MS patients without treatment. ^18^ In patients with RRMS and primary progressive MS (PPMS) with positive B cells for G1m1 (IgG1 heavy chain gene), Lossius *et al*. detected IgG1 ASC with a phenotype compatible with highly proliferating PB (CD19 ^dim^CD27 ^hi^CD38 ^+^) and with high expression of CD138 ^+^, HLA-DR ^+^ and KI67 ^+^ in CSF. ^40^ In pediatric MS, an increase in memory B cells in CSF, with a predominance of non-switched memory B cells and PB, was found, while in adults with MS, class-switched memory B cells and PC predominated in CSF during relapses of MS. ^25, 41^ Using a deep repertoire sequencing of IgG heavy chain variable genes (IgG-Vh) in paired CSF and peripheral blood from patients with MS, VonBudinghen *et al*. found that there was a cluster of clonally related B cells involved in a bidirectional cell exchange across the BBB, with some of them being present primarily in the CNS while others were present in the periphery or in both compartments. ^42^ Additionally, using the same protocol, they found evidence of clonally related B cell receptors in a patient’s blood and CSF, after seven years of therapy with rituximab, indicating a prolonged presence in this compartment during the disease span due to recirculating memory B cells or LLPC. ^43^ Greenfield *et al*. found that clonally related B cells were present as class-switched IgG and CD27 ^+^ in the CSF of patients with MS, leading to the conclusion that, despite having been on DMT, there were complex patterns of persistence of clonal B cells in CSF and blood. ^22^ A significant depletion of CD20 ^+^ B cells has been detected in the blood, with a partial and transient depletion in the CSF and the CNS perivascular spaces, after therapy with rituximab. ^44, 45^ Table 1 presents a summary of the different surface markers that characterize the B cell lineage through its lifespan.

**Table 1.** B cell subsets surface markers. Up-to-date reported B-cell subtypes with reference to the B cell lineage in MS **.** Sub-types from other inflammatory conditions are also mentioned. ^88^ Abbreviations **:** SLO: secondary lymphoid organ; GC: germinal center; BM: bone marrow; ASC: antibody secreting cells; LLPC: long lived plasma cells. ‘Compartment’ makes reference to the organ where the biomarkers were found.

B-cell subtype	Surface biomarkers	Compartment	Reference
**Stem cell**	CD34, HLA-DR	BM	12
**Pro-B cell**	CD19 ^+^CD34 ^+^IgM ^-^	BM	20
	CD34, HLA-DR, CD19	BM	12
**Pre-B cell**	HLA-DR, CD19, CD20, Pre-BCR	BM	12
	CD19 ^+^CD34 ^+^IgM ^-^	BM	20
**Immature B cell**	HLA-DR, CD19, CD20, IgM	BM	12
**Transitional B cells**	CD19 ^+^CD27 ^-^CD38 ^hi^CD24 ^hi^	Blood	27
	HLA-DR, CD19, CD20, IgM, IgD, CD38	Blood	12
	CD19 ^+^IgD ^+^CD27 ^-^CD38 ^+^	Spleen/blood	20
	CD19 ^+^CD38 ^++^CD24 ^++^	Spleen/blood	20
	CD24 ^high^CD38 ^high^	Blood, SLO	85
	CD38 ^++^CD10 ^+^IgD ^+^	Blood	88
**Naïve B cell**	HLA-DR, CD19, CD20, IgM, IgD	SLO/Blood	12
	CD19 ^+^CD27 ^-^	CSF	21
	CD19 ^+^CD27 ^-^IgD ^+^	Blood	27
	CD19 ^+^IgD ^+^CD27 ^+^	Spleen/blood	20
	CD19 ^+^CD27 ^-^IgD ^+^	CSF	96
	CD27 ^-^IgD ^+^	Blood	88
**Non class-switched memory B cell**	CD19 ^+^IgD ^+^CD27 ^+^	Spleen/blood	20
**Pre-class switched memory B cell**	CD27 ^+^IgD ^+^	Blood	88
**Memory B cell**	CD19 ^+^CD27 ^+^IgD ^-^	Blood	27
	HLA-DR, CD19, CD20, CD27	Blood, SLO	12
	CD19 ^+^CD27 ^+^	CSF	21
	CD19 ^+^CD27 ^+^CD80 ^+^CD86 ^+^	CSF	96
**Centroblast**	CD19 ^+^CD38 ^+^CD77 ^+^Ki67 ^+^Bcl-2 ^-^	CSF	96
**Centrocyte**	CD19 ^+^CD38 ^+^CD77 ^-^	CSF	96
**Post-class witched memory B cell**	CD27 ^+^IgD ^-^	Blood	88
**Class-switched Memory B cell**	CD19 ^+^IgD ^-^CD27 ^+^	Spleen/blood	20
**Short lived plasmablast**	CD19 ^+^CD27 ^++^CD138 ^+^CD38 ^+^HLA-DR ^++^	CSF	21
**Plasmablasts**	HLA-DR, CD19, CD20 ^low^, CD27, CD38	SLO/blood	12
	CD19 ^+^CD27 ^+^CD38 ^+^	Blood	27
	CD38 ^++^CD27 ^++^IgD ^-^	Blood	88
	CD19 ^+^CD27 ^+^CD38 ^++^	Spleen/blood	20
	CD19 ^+^CD138 ^++^	Spleen/blood	20
	CD19 ^+^CD27 ^++^CD138 ^+^CD38 ^++^	CSF	21
	CD19 ^dim^CD27 ^hi^CD38 ^+^CD138HLA-DR Ki67	CSF, blood	40
**Plasma cells**	CD19 ^+^CD27 ^++^CD138 ^+^CD38 ^-^	CSF	21
	CD19 ^low^, CD27, CD38, CD138	SLO/blood	12
	CD38 ^+^CD138 ^+^	Spleen/blood	20
	CD19 ^-^CD138 ^+^	CSF	96
	CD19 ^+^CD138 ^+^	Blood	27
**Bregs cell**	IL10, CD27, CD5, CD25, CD86, CD24, CD38	Blood	27
	CD24 ^high^CD27 ^+^	Blood, SLO	85
	CD19 ^+^CD24 ^++^CD38 ^++^	Spleen/blood	20
	CD19 ^+^CD5 ^+^CD1d ^++^	Spleen/blood	20
**Double Negative B cell**	CD27 ^-^IgD ^-^	Blood	88
**LLPC**	CD19 ^-^IgD ^-^CD27 ^+^CD138 ^+^CD38 ^Hi^	BM	101
	CD19 ^-^CD138 ^+^CD38 ^Hi^	BM	101
**ASC in CSF**	CD19 ^-^CD27 ^+^CD38 ^+^IgG	CSF	40
**B cells in CSF**	CD19 ^+^CD138 ^-^	CSF	21
	CD19 ^dim^CD27 ^hi^CD138 ^+^ASC	CSF	40

### Mapping of cell markers is essential to evaluate treatment response

Advances in immunotherapy have made it possible to limit the presence and expansion of B cells, thus reducing relapses and the progression of disability. However, the determination of which cells from the lineage could be responsible for clinical deterioration, or improvement, is still to be investigated. In the treatment of other autoimmune diseases, such as pemphigus, the mapping of cell markers has been used to evaluate the response to treatment, finding alterations in the function of CD19 ^+^CD24 ^hi^CD38 ^hi^ Bregs cells, which are present in significantly higher numbers in patients in an active state compared to patients in the remitting state of RRMS. ^46^ Late antibody-mediated rejection continues to be a problem for patients undergoing kidney transplantation and, for many years, it was believed that tolerance and rejection of transplantation were mediated by T cells. ^47^ However, it was recently shown that a population of Bregs may be playing a deleterious role in transplant immunity, and be responsible for the production of alloantibodies. ^48– 50^ Recently, B cells (CD19 ^+^CD24 ^hi^CD38 ^hi^) dysfunction has been reported in peripheral blood, with decreased production of IL10 in patients with RRMS compared to healthy subjects. ^28^ In turn, these cells have a less stimulatory effect on naïve CD4 ^+^ T cells, which produce IFNγ and tumor necrosis factor α (TNFα). ^28^ Additionally, anti-CD19 monoclonal antibody has no effect on Bregs, which are regulators of EAE extension/expression through the secretion of immunosuppressive cytokines. ^51^ In addition, Chen *et al*. determined the presence of autoreactive CD19 ^+^CD20 ^-^ plasma cells in the CSF of patients with RRMS (20.18%), SPMS (29.58%) and PPMS (31.73%), including patients exposed to DMT. ^51^

### Available therapies affecting the B cell lineage

a. **Interferon B (IFNβ)** acts in the periphery, inducing apoptosis of CD27 ^+^ memory B cells through a mechanism that requires FAS receptor/transmembrane activator and calcium-modulating cyclophilin ligand interactor (TACI) signaling, leading to a specific depletion of these memory B cells (which carry the ability to harbor EBV) and an increase in the CD27 ^-^ cell subtype that contains naïve B cells secreting IL10. ^52^ Furthermore, it was observed that memory B cell depletion was accompanied by a reduction in EBV markers. ^52^ IFNβ leads to the inhibition of leukocyte proliferation and antigen presentation. ^53^ It also changes the cytokine profile towards an anti-inflammatory profile in both peripheral blood and the CNS, and reduces T cell migration by inhibiting the activity of the T cell matrix proteinase. ^53^ IFNβ increases naïve B cells and decreases memory B cells in peripheral blood. ^54^ Ersoy *et al*. showed that IFNβ induces the production of high amounts of IL10 compared to therapy with azathioprine in patients with RRMS. ^55^ A meta-analysis study in patients with MS who received IFNβ showed that there was a lower proportion of Th17 cells in the peripheral CD4 ^+^ T cell pool and a reduction of IL17 and IL23 levels in serum. ^56^
b. **Fingolimod** is a sphingosine-1-phosphate (S1P) modulator that binds to the S1P receptor on lymphocytes, and retains naïve B cells and central memory B cells in lymphoid nodes. ^57^ B cell subsets in the periphery are susceptible to being modified by fingolimod, thus leading to a reduction of memory B cells and an increase in the number of transitional B cells and Bregs in the periphery, ^58^ with an associated increase in the production of IL10. ^32^ Treatment with fingolimod has also been associated with a reduction in the lymphocyte count in peripheral blood and with an increase in the percentage of naïve B cells. ^59^ Fingolimod also causes an increase in DN B cells. ^54^ Fingolimod does not affect the exchange of B cells through the BBB, but it affects the intrathecal clonal expansion, thus inhibiting the activity of the GC. ^60^
c. **Dimethyl fumarate (DMF)** causes long-term lymphopenia through its effect on two genes: it induces NFrf2 (antioxidant effect) and inhibits NFkB which, in turn, induces a change from the Th1 to the Th2 subtype. ^57^ DMF increases the ratio of naïve B cells to memory B cells and increases the number of transitional and IL10 producing B cells. ^32^ DMF increases the percentage of naïve B cells, with a relative reduction in memory B cells and DN B cells. ^54^
d. **Teriflunomide** is a drug that inhibits the dihydroorotate dehydrogenase, thus interfering with the biosynthesis of pyrimidines and leading to a reduced cell proliferation. It can significantly reduce Bregs (CD24 ^+^CD38 ^high^), mature B cells (CD24 ^+^CD38 ^low^) and, to a lesser extent, memory B cells (CD24 ^+^CD38 ^-^) in the peripheral blood of patients with RRMS. ^61^ Yilmaz *et al*. reported a reduction of PC in the peripheral blood of patients with RRMS, who were treated with teriflunomide. ^62^
e. **Natalizumab** blocks the entry of T cells (mainly CD4) into the CNS by neutralizing VLD4 or α4β1 integrins. ^57^ Natalizumab in peripheral blood lowers PB and increases memory B cells. ^54^ Kemmerer *et al*. reported an insignificant increase in the number of B cells and memory B cells in patients treated with natalizumab. In addition, PB were reduced due to a mechanism of natalizumab that alters their traffic through the BBB. Natalizumab decreases the exchange of peripheral and intrathecal B cells, but does not modify their intrathecal clonal expansion and can induce a reduction of OCB production in some cases. ^60, 63^ Traub *et al*. found that natalizumab promotes the activation and proinflammatory differentiation of peripheral B cells in MS. ^64^
f. Although **glatiramer acetate (GA)** is a compound affecting T cells, no effect in the maturation and differentiation of B cells has been detected. ^64^ GA interferes with antigen presentation and promotes switching from the pro-inflammatory Th1 state to an anti-inflammatory Th2 state, on top of inducing CD8 ^+^ T regs cell production. ^57^ The pro-inflammatory pattern, mediated by the secretion of IL6 by peripheral B cells, has been shown to abate and switch to a pattern mediated by IL10-secreting Bregs in MS patients treated with GA. ^65^ However, other studies on the efficacy of GA on B cells in patients with RRMS have reported a reduction in the total numbers of B cells, PB and memory B cells in peripheral blood. ^54, 65, 66^ By reducing the expression of intracellular adhesion molecule (ICAM-3), GA contributes to reducing the migration of B cells to the CNS. ^20^
g. **Rituximab** blocks the CD20 receptor, thus removing pathogenic B cells. ^57^ Although rituximab depletes naïve and memory B cells in the circulation and is not as effective in depleting B cells in tissues, effector and regulatory cells are balanced during cell repopulation after therapy. ^32^ Palanichamy *et al*. found that rituximab induced depletion of memory B cells in blood for up to 12 months. ^67^ A depletion of T cells by more than 50% and B cells by 95%, in CSF, has also been reported after treatment with rituximab in patients with RRMS. ^68^ Using the surface B cell marker CD21 in patients with secondary progressive MS, who received IV rituximab on days 0 and 15, and intrathecal rituximab on day 0, six weeks and twelve months later, Komori *et al*. found a significative reduction in CD21 expression in the serum of patients, suggesting a complete and lasting depletion of B cells, as opposed to an insignificant change in CSF corresponding to an incomplete and transitory depletion of B cells in the CNS compartment. ^69^ In patients with neuromyelitis optica (NMO) seropositive for aquaporin-4, treatment with rituximab was followed by no relapse while their memory B cells were below 0.05% in peripheral blood. ^70^ Hausler *et al*., working on a model of EAE induced by myelin oligodendrocyte glycoprotein (MOG) and another model in naïve mice observed, after treatment with the murine subrogate of rituximab, a persistence of mature B cells in the spleen; an early reconstitution of B cells in the bone marrow and in the spleen before being released into the periphery; and a presence of reactive B cells against myelin when the model included activation of B cells. ^71^ Altogether, these findings suggest that pathogenic B cells were able to persist despite an anti CD20 treatment. ^71^ In addition, they reported a fast depletion of B cells in the peripheral blood, which upon discontinuation of treatment, began to repopulate, proving that cells have different sensitivities to therapy with anti CD20. ^71^
h. **Ocrelizumab** is a humanized monoclonal antibody version of rituximab capable of causing more severe CD19 ^+^ cell depletion than rituximab in patients with rheumatoid arthritis. ^72^ Ocrelizumab is also associated with a very long therapeutic effect, up to 22 months, after the last dose as demonstrated by the RRMS clinical trials OPERA I and OPERA II. ^73^ Recent studies have shown that patients who received treatment with rituximab, or ocrelizumab, for RRMS and NMO for several years, developed hypogammaglobulinemia or a defective recovery of B cells, which could be asymptomatic or could present with bacterial infections or recurrent viral diseases. ^74, 75^ The duration of hypogammaglobulinemia fluctuated between one month and eleven years. ^75^ Marcinno *et al*. recommended that, in patients who receive anti CD20 therapy, the serum levels of IgA, IgG, and IgM should be determined before initiation of treatment, and repeated yearly with special attention to patients who present a drop in IgG and IgM early in the course of therapy, and who should receive protection against tetanus. ^76^
i. **Alemtuzumab** depletes the CD52 marker in B and T cells with very long periods of CD4 T cell depletion. ^57^ Alemtuzumab is able to deplete 70 to 95% of CD4 T cells in active relapsing MS. ^25^ During the reconstitution of B cells after treatment with alemtuzumab, there is a predominance of immature transitional cells, which is followed by a predominance of mature naïve B cells, accompanied by an increase in BAFF, while the reappearance of memory B cells is slow. ^32, 77^ Mohn *et al*. reported a change in the distribution of B cells toward a B cell-naïve phenotype in MS patients treated with alemtuzumab, observing negativization of OCB in two patients. ^78^ The adverse effect of alemtuzumab, including autoimmune disease of the thyroid gland, kidney, platelets and lungs, are well known and correlate with the early recovery of the B cell population with persistence of CD4 T cell depletion, especially during the first year of therapy. ^79^
j. **Atacicept** binds to BAFF and to APRIL, blocking the maturation, differentiation and survival of B lymphocytes. ^57, 80^ Atacicept depletes transitional and naive B cells, PB and PC, and IL10-producing B regs. ^32, 81^ Atacicept causes B cell depletion without affecting progenitor cells (pre- and pro-B cells) and memory B cells. ^54^ Treatment of MS patients with atacicept, unexpectedly, induced more relapses in the ATAMS trial. ^80, 81^
k. **Cladribine** is a chlorinated deoxyadenosine analog, partially resistant to adenosine deaminase. ^34^ The role of cladribine as an immune reconstitution therapy (IRT) has been proven by its prolonged depleting effect on CD4 ^+^ T and B lymphocytes in the periphery. ^82, 83^ Cladribine has the ability to reduce class-switched and unswitched memory B cells to a level comparable to that seen in therapy with alemtuzumab. ^82^
l. **Inebilizumab** is an anti-CD19 ^+^ B cell drug with the ability to deplete the B cell lineage from pro-B cell to PC stage, which was recently reported to induce rapid depletion of B cells and PC in MS patients in a phase I study. ^84^ A new generation of anti-CD20 therapies capable of depleting B cells in the resident organ is under development, including obinutuzumab, ^85^ although it has not been tested in the treatment of MS yet.
m. **Human immunoglobulin G (IVIg)** acts on steady-state B cells, inhibiting the homeostatic proliferation of B cells accompanied by an induction of cell aggregation. ^86^
n. **Autologous haematopoietic stem cell transplantation (AHSCT)** is another alternative treatment that achieves a therapeutic effect by depleting all lymphocytic cell population involved in MS; however, its efficacy depends on the type of cell ablation used, since, as described by Hausler *et al.* while reporting an animal model of EAE, the reconstitution of B cells after anti-CD20 therapy stems from the B cell population that has survived in the bone marrow and spleen. ^71^ An analysis of peripheral blood lymphocyte reconstitution after AHSCT **,** with high-dose immunosuppressive therapy in patients with RRMS followed for two years, disclosed a greater progressive expansion of the population of naïve B cells in the first and second year post-transplant. ^87^ At one month, patients with systemic sclerosis who underwent AHSCT had a transient increase in transitional B cells and PB with an increase in the percentage of naïve B cells up to 14 months; their cytokine profile also changed in the long term, increasing IL10 secretion. ^88^ The B cell compartment also showed decreased percentages of pre- and post-switch memory, as well as DN B cells. ^88^

Burton tyrosine kinase (BTK) inhibitors appear promising as potential therapeutic agents, since evobrutinib has previously been shown to prevent the activation of B cells and improves the clinical course in EAE. ^89^

## Discussion

The origin of MS still remains enigmatic, although different animal models of EAE have been developed, emulating a peripheral attack compromising the CNS, or an intrinsic CNS pathology process with effect in the peripheral blood. ^90^ Sabatino *et al*. have suggested that the paradigm of autoimmune reaction occurring within the CNS may coexist with the outside-in paradigm. ^12^ Either way, B and T cells are interdependent in the pathogenesis of MS. Inside the brain, the TLO found in the meninges are the driving force of the autoimmune pathogenic process. ^91^ It has been proposed that previous EBV infection, vitamin D deficiency, and/or a genetic substrate may be the initiators or determinants of the disease process in MS. The B cell lineage plays a crucial role in the pathogenesis of MS and remains active during the course of the disease, in the periphery and CNS, and an aggressive depletion with current therapies can only control the clinical activity and slow down the progression toward disability. Deciphering the intricate variety of phenotypes and the role of the different B cell subsets in MS would be paramount for a complete understanding of this disease.

The acknowledgement of the role of B cell subsets in the presentation of several inflammatory diseases has stemmed from observations in autoimmune conditions such as rheumatoid arthritis, end-stage renal disease secondary to nephritis, bullous pemphigus, and granulomatosis with polyangiitis. ^92, 93^ Patients with end-stage kidney disease, who have an increase in transitional B cells and Bregs in the blood before transplant, and who present a significant reduction in post-transplant Bregs, are more likely to suffer acute and chronic rejection. ^92^ Although Bregs appear as anti-inflammatory cells, there is evidence that they may play a pro-inflammatory role in certain pathologies. In another study in patients with bullous pemphigus, Liu *et al*. confirmed that identifying the role of each cell subtype in the pathophysiology of the disease is crucial. ^94^ In the same study, a dysfunction of Bregs exhibiting a pro-inflammatory phenotype was observed to contribute to the production of autoantibodies. ^94^

In MS, it has been documented that memory B cells can lead to an exacerbation of RRMS through the activation of T cells in the periphery. ^5^ Furthermore, the fact that memory B cell numbers are decreased under the action of various DMT, and the fact that they were not found to be eliminated by atacicept, confirms their pathogenic role. Most of the immunomodulatory therapies currently available generally induce a reduction in memory B cells and an increase in naïve B cells in peripheral blood, which translates into clinical improvement. In contrast, natalizumab blocks the passage of B cells, mainly memory B cells, through the BBB, increasing their number in peripheral blood. The effect of B cell intrathecally depleting agents is not fully understood.

In relation to Bregs, Matsushita *et al*. observed that depletion of B cells, before the induction of an EAE model, exacerbated the severity of the pathology, due to the depletion of the Bregs population and its suppressive capacity; in contrast, depletion during the acute phase decreased symptoms by affecting the effector cells, which prevented the activation of CD4 ^+^ T cells. ^95^ Identifying the subtypes of B cells which may be responsible for the inflammatory process in MS, in the periphery and in the CNS, is essential to achieve a selective and timely intervention in order to modulate or neutralize their function and to avoid disease progression, without interfering with the functions of immune surveillance and decreasing the anti-inflammatory response of Bregs. ^85, 96^ The ability of some cells of the B lineage to transform into Bregs, counteracting inflammation through the production of IL10, is remarkable and warrants to be considered for the development of better therapeutic strategies. Several studies conducted on patients who received kidney transplantation and patients with other autoimmune diseases, have shown that treatment with anti-CD20 is effective in the restoration of the balance between effector B cell and Bregs, and that the repopulation of B cells might predict a clinical relapse. ^97^

Current consensus dictates that early initiation of therapy in patients with MS leads to a better prognosis. However, a common dilemma in the MS clinics entails deciding when patients with CIS should start treatment. It is usually considered that CIS patients with high risk factors such as presence of OCB, uptake lesions on MRI, and marked severity of the clinical episode are most likely to evolve to clinically definitive MS or RRMS. Another dilemma is observed in patients with CIS who have been started on DMT, based on risk factors, but who, after four or five years of follow up, do not display evidence of disease activity yet. ^98^ Mapping B cell subtypes in peripheral blood and CSF could be considered as an additional tool to determine alterations in the B cell lineage, which could be suggestive of disease activity in these subjects.

We still believe that the meningeal TLO works as an operation center with the ability to magnify an auto-immune response by maintaining antibody diversity, B cell differentiation isotype switching, oligoclonal expansion and local production of autoreactive PC. ^99^ However, recent studies with intrathecal rituximab have shown an inadequate effect in progressive MS and failed to show an early effect, with persistence of markers of inflammation in CSF and leptomeningeal enhancement, in PPMS. ^69^^,^^100^ Factors involved in a decrement of CNS efficacy of intrathecal rituximab include a decreased complement-dependent cytotoxicity (due to a low complement concentration in the CSF), a decreased antibody-dependent cytotoxicity (due to a lower proportion of CD56 ^dim^NK cells) and a poor bioavailability of rituximab for the B cells embedded in the CNS due to the dynamics of the CSF flow from the lumbar cistern to the arachnoid granulations. ^69^

The main goal of this review entailed the summary of the B cell lineage diversity, following the transformation that cells undergo in each specialization stage allowing them to fulfill distinct roles in their attack on the CNS. Simultaneously, it raises the need to give a directed treatment that could improve drug delivery in the CNS, and a more ingenious monitoring of individual responses to therapies, in order to personalize treatment protocols. Finally, the complexity of the function of LLPC and the extraordinary role that they play in the B cell lineage require further investigation, as well as a deeper review of the contemporary medical literature. ^101^

## Conclusion

It is becoming evident that a better identification of the role of different B cell subsets, in the periphery and CNS during the lifespan of MS, will be of paramount significance for the understanding of the pathogenesis of the disease. Specifically, a careful evaluation of the expression of surface markers of transitional, naïve, memory B cells and Bregs, in blood and/or CSF, could contribute to a prompt identification of patients who are not responding to therapy and who may be susceptible to undergo relapses and disease progression. A better understanding of the role of these cell subsets would be useful for engineering intelligent cell therapies that, hopefully, may permit a better control of the disease in the future. This approach would encourage us to rethink the current therapeutic strategy in order to improve the prognosis and quality of life of patients with MS.

## References

[1] Klineova S Lublin FD : Clinical course of multiple sclerosis. *Cold Spring Harbor Perspectives in Medicine.* 2018;8:a028928. 10.1101/cshperspect.a028928 29358317 PMC6120692

[2] Sospedra M : B cells in multiple sclerosis. *Current Opinion in Neurology.* 2018;31:256–262. 10.1097/WCO.000000000000563 29629941

[3] Hohlfeld R Dornmair K Meinl E : The search for the target antigens of multiple sclerosis, part 2: CD8+ T cells, B cells, and antibodies in the focus of reverse-translational research. *Lancet Neurology.* 2016;15:317–331. 10.1016/S1474-4422(15)00313-0 26724102

[4] Li R Patterson KR Bar-Or A : Reassessing B cell contributions in multiple sclerosis. *Nature Immunology.* 2018;19:696–707. 10.1038/s41590-018-0135-x 29925992

[5] Jelcic I Nimer FA Wang J : Memory B cells activate brain-homing, autoreactive CD4 ^+^T cells in multiple sclerosis. *Cell.* 2018;175:85–100.e23. 10.1016/j.cell.2018.08.011 30173916 PMC6191934

[6] Melcher F : Checkpoints that control B cell development. *The Journal of Clinical Investigation.* 2015;125:2203–2210. 10.1172/JCI78083 25938781 PMC4497745

[7] Radbruch A Muehlinghaus G Luger EO : Competence and competition: The challenge of becoming a long lived plasma cell. *Nature Reviews. Immunology.* 2006;6:741–750. 10.1038/nri1886 16977339

[8] Lindquist RL Niesner RA Hauser AE : In the right place, at the right time: spatiotemporal conditions determining plasma cell survival and function. *Frontiers in Immunology.* 2019;10:788. 10.3389/fimmu.2019.00788 31068930 PMC6491733

[9] Chunder R Schropp V Kuerten S : B cells in multiple sclerosis and virus-induced neuroinflammation. *Frontiers in Neurology.* 2020;11:591894. 10.3389/fneur.2020.591894 33224101 PMC7670072

[10] Chu VT Berek C : The establishment of the plasma cell survival niche in the bone marrow. *Immunological Reviews.* 2013;251:177–188. 10.1111/imr.12011 23278749

[11] Roco JA Mesin L Binder SC : Class-switch recombination occurs infrequently in germinal centers. *Immunity.* 2019;51:337–350.e7. 10.1016/j.immuni.2019.07.001 31375460 PMC6914312

[12] Sabatino JJ Jr Probstel AK Zamvil SS : B cells in autoimmune and neurodegenerative central nervous system diseases. *Nature Reviews. Neuroscience.* 2019;20:728–745. 10.1038/s41583-019-0233-2 31712781

[13] Lightman SM Utley A Lee KP : Survival of long-lived plasma cells (LLPC): piecing together the puzzle. *Frontiers in Immunology.* 2019;10:965. 10.3389/fimmu.2019.00965 31130955 PMC6510054

[14] Vinuesa CG Sanz I Cook MC : Dysregulation of germinal centres in autoimmune disease. *Nature Reviews. Immunology.* 2009;9:845–857. 10.1038/nri2637 19935804

[15] Dhaeze T Stinissen P Liston A : Humoral autoimmunity: a failure of regulatory T cells?. *Autoimmunity Reviews.* 2015;14:735–741. 10.1016/j.autrev.2015.04.006 25913138

[16] Lassmann H : Multiple sclerosis pathology. *Cold Spring Harbor Perspectives in Medicine.* 2018;8:a028936. 10.1101/cshperspect.a028936 29358320 PMC5830904

[17] Meinl E Krumbholz M Hohlfeld R : B lineage cells in the inflammatory central nervous system environment: migration, maintenance, local antibody production, and therapeutic modulation. *Annals of Neurology.* 2006;59:880–892. 10.1002/ana.20890 16718690

[18] Corcione A Casazza S Ferretti E : Recapitulation of B cell differentiation in the central nerovus system of patients with multiple sclerosis. *Proc Natl Acad Sci USA.* 2004;101:11064–11069. 10.1073/pnas.0402455101 15263096 PMC503741

[19] Alsughayyir J Pettigrew GJ Motallebzadeh R : Spoiling for a fight: B Lymphocytes as initiator and effector populations within tertiary lymphoid organs in autoimmunity and transplantation. *Frontiers in Immunology.* 2017;8:1639. 10.3389/fimmu.2017.01639 29218052 PMC5703719

[20] Claes N Fraussen J Stinissen P : B cells are multifunctional players in multiple sclerosis pathogenesis: Insights from therapeutic interventions. *Frontiers in immunology.* 2015;6:642. 10.3389/fimmu.2015.00642 26734009 PMC4685142

[21] Cepok S Rosche B Grummel V : Short-lived plasma blasts are the main B cell effector subset during the course of multiple sclerosis. *Brain.* 2005;128:1667–1676. 10.1093/brain/awh486 15800022

[22] Greenfield AL Dandekar R Ramesh A : Longitudinally persistent cerebrospinal fluid B cells can resist treatment in multiple sclerosis. *JCI Insight.* 2019;4(6):e126599. 10.1172/jci.insight.126599 30747723 PMC6482992

[23] Anthony IC Crawford DH Bell JE : B lymphocytes in the normal brain: contrasts with HIV-associated lymphoid infiltrates and lymphomas. *Brain.* 2003;126:1058–1067. 10.1093/brain/awg118 12690046

[24] Louveau A Smirnov I Keyes TJ : Structural and functional features of central nervous system lymphatics. *Nature.* 2015;523:337–341. 10.1038/nature14432 26030524 PMC4506234

[25] Baker D Marta M Pryce G : Memory B cells are major targets for effective immunotherapy in relapsing multiple sclerosis. *eBioMedicine.* 2017;16:41–50. 10.1016/j.ebiom.2017.01.042 28161400 PMC5474520

[26] Peng B Ming Y Yang C : Regulatory B cells: the cutting edge of immune tolerance in kidney transplantation. *Cell Death & Disease.* 2018;9:109. 10.1038/s41419-017-0152-y 29371592 PMC5833552

[27] Blumenfeld S Staun-Ram E Miller A : Fingolimod therapy modulates circulating B cell composition, increases B regulatory subsets and production of IL-10 and TGFβ in patients with multiple sclerosis. *Journal of Autoimmunity.* 2016;70:40–51. 10.1016/j.jaut.2016.03.012 27055778

[28] Cencioni MT Ali R Nicholas R : Defective CD19 ^+^CD24 ^hi^CD38 ^hi^transitional B-cell function in patients with relapsing-remitting MS. *Multiple Sclerosis.* 2021;27:1187–1197. 10.1177/1352458520951536 32924828

[29] Blair PA Noreña LY Flores-Borja F : CD19 ^+^CD24 ^hi^CD38 ^hi^ B cells exhibit regulatory capacity in healthy individuals but are functionally impaired in systemc lupus erythematosus patients. *Immunity.* 2010;32:129–140. 10.1016/j.immuni.2009.11.009 20079667

[30] Geladaris A Hausler D Bruck W : “B cells regulate chronic CNS inflammation in an IL10 dependent manner”. Oral presentation plenary section PS06.05 11 sept 2020. 8 ^th^ joined Actrims-Ectrims meeting. MS virtual 2020. 20079667

[31] Carter NA Vasconcellos R Rosser EC : Mice lacking endogenous Il-10-producing regulatory B cells develop exarcebated disease and present with an increased frequency of Th1/Th17 but a decrease in regulatory T cells. *Journal of Immunology.* 2011;186:5569–5579. 10.4049/jimmunol.1100284 21464089

[32] Staun-Ram E Miller A : Effector and regulatory B cells in multiple sclerosis. *Clinical Immunology.* 2017;184:11–25. 10.1016/j.clim.2017.04.014 28461106

[33] Heine G Drozdenko G Grün JR : Autocrine IL-10 promotes human B-cell differentiation into IgM- or IgG- secreting plasmablasts. *European Journal of Immunology.* 2014;44:1615–1621. 10.1002/eji.201343822 24643722

[34] Magliozzi R Howell OW Nicholas R : Inflammatory intrathecal profiles and cortical damage in multiple sclerosis. *Annals of Neurology.* 2018;83:739–755. 10.1002/ana.25197 29518260

[35] Thi Cuc B Pohar J Fillatreau S : Understanding regulatory B cells in autoimmune diseases: the case of multiple sclerosis. *Current Opinion in Immunology.* 2019;61:26–32. 10.1016/j.coi.2019.07.007 31445312

[36] Farian G Magliozzi R Pitteri M : Increased cortical lesion load and intrathecal inflammation is associated with oligoclonal bands in multiple sclerosis: a combined CSF and MRI study. *Journal of Neuroinflammation.* 2017;14:40. 10.1186/s12974-017-0812-y 28222766 PMC5319028

[37] Machado-Santos J Saji E Tröscher AR : The compartmentalized inflammatory response in the multiple sclerosis brain is composed of tissue-resident CD8+ T lymphocytes and B cells. *Brain.* 2018;141:2066–2082. 10.1093/brain/awy151 29873694 PMC6022681

[38] Claes N Fraussen J Vanheusden M : Age-associated B cells with proinflammatory characteristics are expanded in a proportion of multiple sclerosis patients. *Journal of Immunology.* 2016;197:4576–4583. 10.4049/jimmunol.1502448 27837111

[39] Fraussen J Marquez S Takata K : Phenotypic and Ig repertoire analyses indicate a common origin of IgD ^-^CD27 ^-^ double negative B cells in healthy individuals and multiple sclerosis patients. *Journal of Immunology.* 2019;203:1650–1664. 10.4049/jimmunol.1801236 31391234 PMC6736705

[40] Lossius A Tomescu-Baciu A Holmøy T : Selective intrathecal enrichment of G1m1-positive B cells in multiple sclerosis. *Annals of Clinical Translational Neurology.* 2017;4:756–761. 10.1002/acn3.451 29046884 PMC5634349

[41] Schwarz A Balint B Korporal-Kuhnke M : B cell populations discriminate between pediatric- and adult-onset multiple sclerosis. *Neurol Neuroimmunol Neuroinflamm.* 2017;4(1):e309. 10.1212/NXI.0000000000000309 28053999 PMC5182056

[42] Von Büdinghen HC Kuo TC Sirota M : B cell exchange across the blood brain barrier in multiple sclerosis. *The Journal of Clinical Investigation.* 2012;122:4533–4543. 10.1172/JCI63842 23160197 PMC3533544

[43] Von Büdinghen HC Bischof A Eggers EL : Onset of secondary progressive MS after long-term rituximab therapy - a case report. *Annals of Clinical Translational Neurology.* 2017;4:46–52. 10.1002/acn3.377 28078314 PMC5221476

[44] Cross AH Stark JL Lauber J : Rituximab reduces B cells and T cells in cerebrospinal fluid of multiple sclerosis patients. *Journal of Neuroimmunology.* 2006;180:63–70. 10.1016/j.jneuroim.2006.06.029 16904756 PMC1769354

[45] Martin Mdel P Cravens PD Winger R : Depletion of B lymphocytes from cerebral perivascular spaces by rituximab. *Archives of Neurology.* 2009;66(8):1016–1020. 10.1001/archneurol.2009.157 19667224

[46] Zhu HQ XU RC Chen YY : Impaired function of CD19(+)CD24(hi)CD38(hi) regulatory B cells in patients with pemphigus. *The British Journal of Dermatology.* 2015;172:101–110. 10.1111/bjd.13192 24935080

[47] Nouël A Simon Q Jamin C : Regulatory B cells: an exciting target for future therapeutics in transplantation. *Frontiers in Immunology.* 2014;5:11. 10.3389/fimmu.2014.00011.eCollection2014 24478776 PMC3897876

[48] Carreras-Planella L Borras FE Franquesa M : Tolerance in kidney transplantation: what is on the B side?. *Mediators of Inflammation.* 2016;2016:8491956. 10.1155/2016/8491956 27956762 PMC5121468

[49] Chong AS Khiew SH : Transplantation tolerance: don’t forget about the B cells. *Clinical and Experimental Immunology.* 2017;189:171–180. 10.1111/cei.12927 28100001 PMC5508319

[50] Karahan GE Claas FH Heidt S : B cell immunity in solid organ transplantation. *Frontiers in Immunology.* 2016;7:686. 10.3389/fimmu.2016.00686 28119695 PMC5222792

[51] Chen D Ireland SJ Davis LS : Autoreactive CD19 ^+^CD20 ^-^ plasma cells contribute to disease severity of experimental autoimmune encephalomyelitis. *Journal of Immunology.* 2016;196:1541–1549. 10.4049/jimmunol.1501376 26764035

[52] Rizzo F Giacomini E Mechelli R : Interferon-β therapy specifically reduces pathogenic memory B cells in multiple sclerosis patients by inducing a FAS-mediated apoptosis. *Immunology and Cell Biology.* 2016;94:886–894. 10.1038/icb.2016.55 27265253

[53] Yong VW Chabot S Stuve O : Interferon beta in the treatment of multiple sclerosis: mechanisms of action. *Neurology.* 1998;51:682–689. 10.1212/wnl.51.3.682 9748010

[54] Kemmerer CL Pernpeintner V Ruschil C : Differential effects of disease modifying drugs on peripheral blood B cell subsets: A cross sectional study in multiple sclerosis patients treated with interferon-β, glatiramer acetate, dimethyl fumarate, fingolimod or natalizumab. *Plos One.* 2020;15:e0235449. 10.1371/journal.pone.0235449 32716916 PMC7384624

[55] Ersoy E Kus CNS Sener U : The effects of interferon-beta on interleukin-10 in multiple sclerosis patients. *European Journal of Neurology.* 2005;12:208–211. 10.1111/j.1468-1331.2004.00986.x 15693810

[56] Hou M Li Y He L : Effect of interferon-Beta treatment on the proportion of T helper 17 cells and related cytokines in multiple sclerosis: A meta-analysis. *Journal of Interferon & Cytokine Research.* 2019;39:771–779. 10.1089/jir.2019.0065 31517556

[57] Martin R Sospedra M Rosito M : Current multiple sclerosis treatments have improved our understanding of MS autoimmune pathogenesis. *European Journal of Immunology.* 2016;46:2078–2090. 10.1002/eji.201646485 27467894

[58] Blumenfeld-Kan S Staun-Ram E Miller A : Fingolimod reduces CXCR4-mediated B cell migration and induces regulatory B cell-mediated anti-inflammatory immune repertoire. *Multiple Sclerosis and Related Disorders.* 2019;34:29–37. 10.1016/j.msard.2019.06.016 31228713

[59] Mills EA Mao-Draayer Y : Aging and lymphocyte changes by immunomodulatory therapies impact PML risk in multiple sclerosis patients. *Multiple Sclerosis.* 2018;24:1014–1022. 10.1177/1352458518775550 29774781 PMC6013383

[60] Kowarik MC Astling D Lepennetier G : Differential effects of fingolimod and natalizumab on B cell repertoires in multiple sclerosis patients. *Neurotherapeutics.* 2021;18:364–377. 10.1007/s13311-020-00975-7 33258072 PMC8116403

[61] Gandoglia I Ivaldi F Laroni A : Teriflunomide treatment reduces B cells in patients with MS. *Neurol Neuroimmunol Neuroinflamm.* 2017;4:e403. 10.1212/NXI.0000000000000403 29082295 PMC5656406

[62] Yilmaz V Ulusoy C Hajtovic S : Effects of teriflunomide on B cell subsets in MuSK-induced experimental autoimmune myasthenia gravis and multiple sclerosis. *Immunological Investigations.* 2020;50:671–684. 10.1080/08820139.2020.1785491 32597289

[63] Harrer A Tumani H Niendorf S : Cerebrospinal fluid parameters of B cell-related activity in patients with active disease during natalizumab therapy. *Multiple Sclerosis.* 2013;19:1209–1212. 10.1177/1352458512463483 23093485

[64] Traub JW Pellkofer HL Grondey K : Natalizumab promotes activation and pro-inflammatory differentiation of peripheral B cells in multiple sclerosis patients. *Journal of neuroinflammation.* 2019;16:228. 10.1186/s12974-019-1593-2 31733652 PMC6858649

[65] Ireland SJ Guzman AA : O´Brien DE, The effect of glatiramer acetate therapy on functional properties of B cells from patients with relapsing-remitting multiple sclerosis. *JAMA Neurology.* 2014;71(11):1421–1428. 10.1001/jamaneurol.2014.1472 25264704 PMC4335670

[66] Kuerten S Jackson LJ Kaye J : Impact of glatiramer acetate on B cell-mediated pathogenesis of multiple sclerosis. *CNS Drugs.* 2018;32(11):1039–1051. 10.1007/s40263-018-0567-8 30315499 PMC6223706

[67] Palanichany A Jahn S Nickles D : Rituximab efficiently depletes increased CD20-expressing T cells in multiple sclerosis patients. *Journal of Immunology.* 2014;193:580–586. 10.4049/jimmunol.1400118 PMC4082756 24928997

[68] Piccio L Naismith RT Trinkaus K : Changes in B- and T- lymphocyte and chemokine levels with rituximab treatment in multiple sclerosis. *Archives of Neurology.* 2010;67:707–714. 10.1001/archneurol.2010.99 20558389 PMC2918395

[69] Komori M Lin YC Cortese I : Insufficient disease inhibition by intrathecal rituximab in progressive multiple sclerosis. *Annals of Clinical Translational Neurology.* 2016;3:166–179. 10.1002/acn3.293 27042677 PMC4774261

[70] Kim SH Kim W Li XF : Repeated treatment with rituximab based on the assessment of peripheral circulating memory B cells in patients with relapsing neuromyelitis optica over 2 years. *Archives of Neurology.* 2011;68:1412–1420. 10.1001/archneurol.2011.154 21747007

[71] Hausler D Hausser-Kinzel S Feldmann L : Functional characterization of reappearing B cells after anti-CD20 treatment of CNS autoimmune disease. *Proc Natl Acad Sci USA.* 2018;115:9773–9778. 10.1073/pnas.1810470115 30194232 PMC6166805

[72] Genovese MC Kaine JL Lowenstein MB : Ocrelizumab, a humanized anti-CD20 monoclonal antibody, in the treatment of patients with rheumatoid arthritis. A phase I/II randomized, blinded, placebo-controlled, dose-ranging study. *Arthritis and Rheumatism.* 2008;58:2652–2661. 10.1002/art.23732 18759293

[73] Hausser SL Bar-Or A Comi G : Ocrelizumab versus interferon Beta-1a in relapsing multiple sclerosis. *The New England Journal of Medicine.* 2017;376:221–234. 10.1056/NEJMoa1601277 28002679

[74] Sacco KA Abraham RS : Consequences of B-cell-depleting therapy: hypogammaglobulinemia and impaired B-cell reconstitution. *Immunotherapy.* 2018;10:713–728. 10.2217/imt-2017-0178 29569510

[75] Vollmer BL Wallach AI Corboy JR : Serious safety events in rituximab-treated multiple sclerosis and related disorders. *Annals of Clinical Translational Neurology.* 2020;7(9):1477–1487. 10.1002/acn3.51136 32767531 PMC7480911

[76] Marcinno A Marnetto F Valentino P : Rituximab-induced hypogammaglobulinemia in patients with neuromyelitis optica spectrum disorders. *Neurol Neuroimmunol Neuroinflamm.* 2018;5:e498. 10.1212/NXI.0000000000000498 30258855 PMC6148550

[77] Thompson SAJ Jones JL Cox AL : B-cell reconstitution and BAFF after alemtuzumab (Campath-1H) treatment of multiple sclerosis. *Journal of Clinical Immunology.* 2010;30:99–105. 10.1007/s10875-009-9327-3 19763798

[78] Möhn N Pfeuffer S Ruck T : Alemtuzumab therapy changes immunoglobulin levels in peripheral blodd and CSF. *Neurol Neuroimmunol Neuroinflamm.* 2020;7:e654. 10.1212/NXI.0000000000000654 31826986 PMC7007635

[79] Devonshire V Phillips R Wass H : Monitoring and management of autoimmunity in multiple sclerosis patients treated with alemtuzumab: practical recommendations. *Journal of neurology Supplement to.* 2018;265:2494–2505. 10.1007/s00415-018-8822-y 29525836 PMC6182701

[80] Kappos L Hartung HP Freedman MS : Atacicept in multiple sclerosis (ATAMS): a randomized, placebo-controlled, double-blind, phase 2 trial. *Lancet Neurology.* 2014;13:353–363. 10.1016/S1474-4422(14)70028-6 24613349

[81] Li R Patterson KR Bar-Or A : Reassessing B cell contributions in multiple sclerosis. *Nature Immunology.* 2018;19:696–707. 10.1038/s41590-018-0135-x 29925992

[82] Ceronie B Jacobs BM Baker D : Cladribine treatment of multiple sclerosis is associated with depletion of memory B cells. *Journal of Neurology.* 2018;265:1199–1209. 10.1007/s00415-018-8830-y 29550884 PMC5937883

[83] Comi G Cook S Giovannoni G : Effect of cladribine tablets on lymphocyte reduction and repopulation dynamics in patients with relapsing multiple sclerosis. *Multiple Sclerosis and Related Disorders.* 2019;29:168–174. 10.1016/j.msard.2019.01.038 30885375

[84] Agius MA Klodowska-Duda G Maciejowski M : Safety and tolerability of inebilizumab (MEDI-551), an anti-CD19 monoclonal antibody, in patients with relapsing forms of multiple sclerosis: results from a phase 1 randomised, placebo-controlled, escalating intravenous and subcutaneous dose study. *Multiple Sclerosis.* 2019;25:235–245. 10.1177/1352458517740641 29143550 PMC6360486

[85] Musette P Bouaziz JD : B cell modulation strategies in autoimmune diseases: new concepts. *Frontiers in Immunology.* 2018;9:622. 10.3389/fimmu.2018.00622 29706952 PMC5908887

[86] Hori A Fujimura T Kawamoto S : Anti-inflammatory intravenous immunoglobulin (IVIg) suppresses homeostatic proliferation of B cells. *Cytotechnology.* 2018;70:921–927. 10.1007/s10616-017-0176-2 29611058 PMC6021294

[87] Karnell FG Lin D Motley S : Reconstitution of immune cell populations in multiple sclerosis patients after autologous stem cell transplantation. *Clinical and Experimental Immunology.* 2017;189:268–278. 10.1111/cei.12985 28498568 PMC5543487

[88] Gernert M Tony HP Schwaneck EC : Autologous hematopoietic stem cell transplantation in systemic sclerosis induces long-lasting changes in B cell homeostasis toward an anti-inflammatory B cell cytokine pattern. *Arthritis Research & Therapy.* 2019;21:106. 10.1186/s13075-019-1889-8 31036055 PMC6489316

[89] Torke S Pretzsch R Häusler D : Inhibition of Bruton´s tyrosine kinase interferes with pathogenic B-cell development in inflammatory CNS demyelinating disease. *Acta Neuropathologica.* 2020;140:535–548. 10.1007/s00401-020-02204-z 32761407 PMC7498502

[90] Nathoo N Yong VW Dunn JF : Understanding disease processes in multiple sclerosis through magnetic resonance imaging studies in animal models. *Neuroimage Clin.* 2014;4:743–756. 10.1016/j.nicl.2014.04.011 24936425 PMC4053634

[91] Negron A Stüve O Forsthuber TG : Ectopic lymphoid follicles in multiple sclerosis: centers for disease control?. *Frontiers in Neurology.* 2020;11:607766. 10.3389/fneur.2020.607766 33363512 PMC7753025

[92] Laguna-Goya R Utrero-Rico A Cano-Romero FL : Imbalance favoring follicular helper T cells over IL10 ^+^ regulatory B cells is detrimental for the kidney allograft. *Kidney International.* 2020;98:732–743. 10.1016/j.kint.2020.02.039 32495741

[93] Bergantini L d’Alessandro M Cameli P : Effects of rituximab therapy on B cell differentiation and depletion. *Clinical Rheumatology.* 2020;39:1415–1421. 10.1007/s10067-020-04996-7 32088800

[94] Liu Z Dang E Li B : Dysfunction of CD19 ^+^CD24 ^hi^CD27 ^+^ B regulatory cells in patients with bullous pemphigoid. *Scientific Reports.* 2018;8:703. 10.1038/s41598-018-19226-z 29335495 PMC5768798

[95] Matsushita T Yanaba K Bouaziz JD : Regulatory B cells inhibit EAE initiation in mice while other B cells promote disease progression. *The Journal of Clinical Investigation.* 2008;118:3420–3430. 10.1172/JCI36030 18802481 PMC2542851

[96] Uccelli A Aloisi F Pistoia V : Unveiling the enigma of the CNS as a B cell fostering environment. *Trends in Immunology.* 2005;26:254–259. 10.1016/j.it.2005.02.009 15866238

[97] Oleinika K Mauri C Salama AD : Effector and regulatory B cells in immune-mediated kidney disease. *Nature Reviews. Nephrology.* 2019;15:11–26. 10.1038/s41581-018-0074-7 30443016

[98] Monschein T Salhofer-Polanyi S Altmann P : Should I stop or should I go on? Disease modifying therapy after the first clinical episode of multiple sclerosis. *Journal of Neurology.* 2021;268:1247–1253. 10.1007/s00415-020-10074-4 32929591 PMC7990829

[99] Londoño AC Mora CA : Role of CXCL13 in the formation of the meningeal tertiary lymphoid organ in multiple sclerosis. *F1000Research.* 2018;7:514. 10.12688/f1000research.14556.3 30345018 PMC6171727

[100] Bonnan M Ferrari S Courtade H : No Early Effect of Intrathecal Rituximab in Progressive Multiple Sclerosis (EFFRITE Clinical Trial). *Mult. Scler. Int.* 2021;2021:8813498. 10.1155/2021/8813498 33763241 PMC7964121

[101] Halliley JL Tipton C Liesveld J : Long lived plasma cells are contained within the CD19 ^-^CD38 ^hi^CD138 ^+^ subset in human bone marrow. *Immunity.* 2015;43:132–145. 10.1016/j.immuni.2015.06.016 26187412 PMC4680845

