# Supplementary material for: Continued dysregulation of the B cell lineage promotes multiple sclerosis activity despite disease modifying therapies
Source: F1000Res. 2023 Aug 1;10:1305. Originally published 2021 Dec 22. [Version 3] doi: 10.12688/f1000research.74506.3 (PMC10467621; doi:10.12688/f1000research.74506.3)
Supplement: Peer review history

# Peer review: Reviewer response for version 3

**DOI:** 10.5256/f1000research.153768.r193018

**Published:** 2023-08-29

**Authors:** Maria Teresa Cencioni

**Affiliations:** 1 Division of Neurology, Department of Brain Sciences, Imperial College London, London, England, UK

I have read the revised manuscript and I am satisfied with the revision. The authors have revised the manuscript addressing my comments and I agree with the indexing.

Is the review written in accessible language?

Yes

Are all factual statements correct and adequately supported by citations?

Yes

Are the conclusions drawn appropriate in the context of the current research literature?

Yes

Is the topic of the review discussed comprehensively in the context of the current literature?

Yes

Reviewer Expertise:

NA

I confirm that I have read this submission and believe that I have an appropriate level of expertise to confirm that it is of an acceptable scientific standard.

# Peer review: Reviewer response for version 2

**DOI:** 10.5256/f1000research.136950.r177504

**Published:** 2023-07-11

**Authors:** Maria Teresa Cencioni

**Affiliations:** 1 Division of Neurology, Department of Brain Sciences, Imperial College London, London, England, UK

The immune system plays an essential role in the pathogenesis of multiple sclerosis. Recent publications reporting the outcomes from clinical trials and DMT have contributed to improving our understanding of the role of B and T cells in MS even though the mechanism underlying the disease is still unknown. Although the relevance of the topic, I have some concerns about the originality of the review as is similar in contents to other reviews published in other journals. 

 Minor revision:

1. It is not clear in which type of MS relapsing-remitting or progressive this type of investigation can improve the disease treatments. The generation of ectopic follicles is associated with a secondary progressive disease where the inflammation compartmentalizes in the leptomeninges. In this stage, inflammation fuels the neurodegeneration processes and treatment-depleting B cells can reduce inflammation but not block the progression of the disease. I would suggest introducing a paragraph regarding the pathology of MS and clarifying at which stage of the disease this investigation can help in selecting a personalised treatment in patients with MS.
2. As the therapies affect the B cell lineage, I would suggest adding also studies comparing the effectiveness of different DMTs and the modulation of the B cell compartment, if it has been investigated. If not, it could be suggested in the discussion as further investigations.
3. In addition, it would be good to also discuss the effectiveness of each treatment in terms of disease activity and progression of disease in MS patients.
4. (The second sentence in the introduction) Could you please explain which other biomarkers in the cerebrospinal fluid have been used for MS diagnosis? Which of them are specific to B cells and in which stage of MS disease? Please add references.
5. Autologous haematopoietic stem cell transplantation: please add the publication.
  Von Niederhäusern V et al.  B-cell reconstitution after autologous haematopoietic stem cell transplantation in multiple sclerosis. Neurol Neuroimmunol Neuroinflamm. 2022.

Is the review written in accessible language?

Yes

Are all factual statements correct and adequately supported by citations?

Yes

Are the conclusions drawn appropriate in the context of the current research literature?

Yes

Is the topic of the review discussed comprehensively in the context of the current literature?

Yes

Reviewer Expertise:

Immunology and neurology

I confirm that I have read this submission and believe that I have an appropriate level of expertise to confirm that it is of an acceptable scientific standard, however I have significant reservations, as outlined above.

## References

[1] : B-Cell Reconstitution After Autologous Hematopoietic Stem Cell Transplantation in Multiple Sclerosis. *Neurol Neuroimmunol Neuroinflamm* .2022;9(6) : 10.1212/NXI.0000000000200027 10.1212/NXI.0000000000200027 36229189PMC9562041

# Response

**Published:** 2023-07-27

**Authors:** Carlos Mora

**Affiliations:** Medicine, Carilion Clinic, Roanoke, Virginia, USA

1. We thank Dr. Cencioni, manuscript reviewer, for her valuable comments to the second version of this article.  Although our review, at first glance, might give the impression that it establishes a parallel with other reviews on the topic of the role of the B-cell lineage in multiple sclerosis, published in other journals, we believe that this review brings specific recognition to the role of the regulatory B cells including naïve, transitional and memory B cells, as well as the role of double negative B cells and CD21 low cells, in the pathogenesis of multiple sclerosis. A better understanding of the role of these cells will be necessary to establish biomarkers for diagnosis, follow-up and prognosis in relapsing-remitting multiple sclerosis (RRMS) and in the chronic progressive forms of the disease.
2. We agree with the reviewer on the fact that treatment–depleting B cells can reduce inflammation, but they cannot block the progression of the disease.  However, in the section titled ‘ *Available therapies affecting the B-cell lineage*’ we also described the effects that at least six of the well-known disease modifying therapies, approved for the treatment of relapsing-remitting multiple sclerosis, have on the B-cell subtypes, on top of the effects that the aggressive B-cell depleting drugs (such as rituximab, ocrelizumab, alemtuzumab, inebilizumab and cladribine) have on the B cell lineage subtypes. Certainly, more studies are needed to understand the B-cell dysregulation in MS, so that we can decipher the mechanisms to control the deleterious effect that B cells exert on the CNS once they establish themselves at the ectopic follicles in the tertiary lymphoid organs. 
3. We modified the first paragraph of the section titled ‘ *B-cell lineage*’ by separating its final segment and creating a new paragraph titled ‘Physiopathology of multiple sclerosis’ since we believe the content of this segment is more akin to be displayed under that sub-title.
4. Following the request of the reviewer, and in order to provide clarification to the content of the second sentence in the introduction paragraph, we modified its content as follows: ‘ *Current MS diagnostic methodologies are based on criteria that include clinical presentation, determination of oligoclonal bands (OCB) and other biomarkers such as the IgG index and IgG synthesis in 24 hours in the cerebrospinal fluid (CSF), as well as presence of inflammatory and/or demyelinating lesions in the magnetic resonance imaging (MRI) analysis*’.
5. In the ‘Discussion’ section, we are adding a new reference in relation to the presence of biomarkers of disease in the spinal fluid [Zhang et al, reference 100]. Accordingly, the following sentences were added to the text: *‘Novel diagnostic and prognostic biomarkers in MS, including determination of the kappa free light chain (KFLC) and KFLC index in CSF, have shown to be promising tools for diagnosis, especially taking into consideration that, in clinical practice, KFLC index might replace OCB as a first line biomarker of disease and it has been shown to predict the evolution from CIS to RRMS [Zhang et al, reference 100].  Another biomarker, chemokine (C-X-C motif) ligand 13 (CXCL13) may be used in the diagnosis of MS in clinical practice for assessment of drug treatment response and disease progression [references 99-100]’*.
6. With reference to the effect of autologous hematopoietic stem cell transplantation (AHSCT) on B-cell reconstitution in patients with MS, we expanded our comments and included the reference recommended by the reviewer [von Niederhäusern et al, reference 89] as follows: ‘ *In a study conducted by Niederhäusern et al, memory B-cell subpopulations were found to recover slowly and to remain below normal levels with a reduced repertoire diversity one year after AHSCT. A successful shifting of B-cell populations, from a predominantly transitional to a naïve immune phenotype, was seen as cells began to recover within 3 months and increased above normal levels 12 months after AHSCT. Memory B-cells that had survived DMT were mainly class switched and persisted in the early posttransplant stage suggesting that not only plasma cells (PC) but also memory subsets had survived conditioning.* *The patients in the study received AHSCT with drugs of the BEAM regimen (BCNU, melphalan, and to a limited extent also etoposide and cytarabine) that can penetrate the CNS compartment, but it remained unknown whether tissue-resident B cells were affected by the conditioning regimen of the transplant* [reference 89].
7. Table 1: The subtitle for Table 1 was modified so that the table is presented as ‘ *B cell subsets surface markers and corresponding compartments in MS*.’ We believe this addition will encourage the readers to match reported B-cell subtypes with the mentioned biomarkers and the compartments where they were found, with the corresponding reference.

# Peer review: Reviewer response for version 2

**DOI:** 10.5256/f1000research.136950.r146792

**Published:** 2022-08-17

**Authors:** Mickaël Bonnan

**Affiliations:** 1 Service de Neurologie, Centre Hospitalier de Pau, Pau, France

We agree with the responses given by authors.

 We regret that the diversity of combined surface markers provided in Table 1 was not commented (we are still not sure that each combination was a different subtype).

 Although we are not convinced by the conclusion stating that identification of B-cell subtypes will help to predict drug/relapse/progression, the article is well written and provides a large review concerning B cell roles in MS.

Is the review written in accessible language?

Yes

Are all factual statements correct and adequately supported by citations?

Yes

Are the conclusions drawn appropriate in the context of the current research literature?

Yes

Is the topic of the review discussed comprehensively in the context of the current literature?

Yes

Reviewer Expertise:

MS, NMOSD

I confirm that I have read this submission and believe that I have an appropriate level of expertise to confirm that it is of an acceptable scientific standard.

# Peer review: Reviewer response for version 1

**DOI:** 10.5256/f1000research.78270.r135362

**Published:** 2022-06-09

**Authors:** Mickaël Bonnan

**Affiliations:** 1 Service de Neurologie, Centre Hospitalier de Pau, Pau, France

This review article deals with the variety and roles of B cells subtypes in MS pathology. Authors tried to engulf diverse data concerning B cell lineage, in general and throughout compartments and MS stages. They finally examined how B cell lineages are affected by disease-modifying treatments (DMT).

 Although this review provides a general overview of the literature concerning B cell lineage in MS, important points were not expanded. Tertiary lymphoid organs (TLO), which drive cortical pathology and prognosis are shortly described. Mutual exchange of cells across the BBB, local heterogeneity of B cell clusters in the brain, location of affinity maturation are too shortly described.

 Our main criticism is nested in figure 2: how consistent and reproducible are data obtained concerning B cell lineages according to the variety of definitions used to qualify each state of maturation?

 *Figure 1: plasmablasts and plasmocytes are only depicted in CSF. Homing of these cells was also described in brain and TLO (i.e. ref 16). Moreover it is easy to evaluate that rare floating ASC (cf ref 21) cannot account of the amount of intrathecal IgG synthesis (they account in a range of ~1%). CSR may also occur in brain TLO. Lastly, bidirectional exchange of B cells, which is major process (e.g. ref 42), was not emphasized in the figure.

 *Sentence: ‘They are identified in perivascular spaces, demyelinating lesions in the brain and spinal cord, and disperse in the meninges where they can form aggregates known as tertiary lymphoid organs (TLO)’. TLO are real and complete lymphoid organs, lacking a conjunctive external capsule and occurring in a non-genetically driven location. Memory B cells are only part of TLO, which are mostly driven by CD3−CD4+CD45+ lymphoid tissue inducer (LTi) cells then stromal cells.

 *Sentence: ‘A significant depletion of CD20+ B cells has been detected in the blood, CSF and perivascular spaces in the CNS after therapy with rituximab and ocrelizumab’. Indeed, B-cells are totally depleted from blood after antiCD20 infusion, whereas CSF compartment remains partly and transiently depleted. This is the point: antiCD20 fails to deplete intrathecal compartment from CD20+ cells, possibly due to the lack of effectors (low complement concentration in CSF, rare NK cells). Intrathecal infusion, although increasing bioavailability in CSF, does not add any efficacy.

 *Table 2. We did not understood the importance of Table 2, except giving example that stringent definition of cell classes is not as stringent as it could be among authors and papers. This table may suggest that surface markers of B cells in MS could be specific. We do not understand the column 'compartment': does it mean that the lineage was found in this compartment? If so, it is worth to also indicate which lineage was NOT found in each compartment.

 *In Conclusion. Sentence: ‘Specifically, a careful evaluation of the expression of surface markers of transitional, naïve, memory B cells and Bregs, in blood and/or CSF, could contribute to a prompt identification of patients who are not responding to therapy and who may be susceptible to undergo relapses and disease progression.‘ Although a real improvement of CSF FACS availability remains possible, we do not believe that the study of B cells will drive therapeutic opportunities or help to monitor the disease. CSF is especially difficult to obtain and is probably not a target for scheduled biological tests. Moreover, as the authors demonstrated throughout the text, the precise role of each B cell subtype is far from being understood in MS.

Is the review written in accessible language?

Yes

Are all factual statements correct and adequately supported by citations?

Yes

Are the conclusions drawn appropriate in the context of the current research literature?

Yes

Is the topic of the review discussed comprehensively in the context of the current literature?

Yes

Reviewer Expertise:

MS, NMOSD

I confirm that I have read this submission and believe that I have an appropriate level of expertise to confirm that it is of an acceptable scientific standard, however I have significant reservations, as outlined above.

# Response

**Published:** 2022-08-01

**Authors:** Carlos Mora

**Affiliations:** Medicine, Carilion Clinic, Roanoke, Virginia, USA

We thank Dr. Bonnan (article reviewer) for his thorough comments to the first version of our manuscript. We have reviewed and modified **Figure 1** to remark that the natural exchange of B-cells across the BBB is bidirectional, that class switch recombination (CSR) may also occur in the brain TLO, and that plasmablasts and plasma cells figure out in the brain (including the TLO), CSF and peripheral blood. We also labelled the T-cells present in the white matter lesion and in the secondary lymphoid organ (SLO).

 In the section titled ‘ **B cells in the CSF compartment in MS**’ the sentence remarked by the reviewer has been modified as follows: ‘ *A significant depletion of CD20+ B cells has been detected in the blood, with a partial and transient depletion in the CSF and the CNS perivascular spaces, after therapy with rituximab*.’

 In the penultimate paragraph of the ‘ **Discussion**’ section, we have added two new references to the manuscript, which give more detailed information about the relevance of the tertiary lymph organ (TLO [ *Londoño AC and Mora CA. Role of CXCL13 in the formation of the meningeal tertiary lymphoid organ in multiple sclerosis. F1000Research 2018, 7:514 https://doi.org/10.12688/f1000research.14556.3*] now reference 99) and about the controversial poor response to therapy with rituximab in the CSF compartment with persistence of B cells ( *Bonnan M, Ferrari S, Courtade H, Money P, Desblache P, Barroso B, Debeugny S. No Early Effect of Intrathecal Rituximab in Progressive Multiple Sclerosis (EFFRITE Clinical Trial). Mult. Scler. Int. 2021, 2021, 8813498 -now reference 100*). This paragraph reads as follows: ‘ *We still believe that the meningeal TLO works as an operation center with the ability to magnify an auto-immune response by maintaining antibody diversity, B cell differentiation isotype switching, oligoclonal expansion and local production of autoreactive PC*. ^99^ *However, recent studies with intrathecal rituximab have shown an inadequate effect in progressive MS and failed to show an early effect, with persistence of markers of inflammation in CSF and leptomeningeal enhancement, in PPMS*. ^69,100^ *Factors involved in a decrement of CNS efficacy of intrathecal rituximab include a decreased complement-dependent cytotoxicity (due to a low complement concentration in the CSF), a decreased antibody-dependent cytotoxicity (due to a lower proportion of CD56 ^dim^NK cells) and a poor bioavailability of rituximab for the B cells embedded in the CNS due to the dynamics of the CSF flow from the lumbar cistern to the arachnoid granulations*. ^69^’

 The first sentence of the ‘ **Conclusion**’ section has been modified as follows: ‘ *It is becoming evident that a better identification of the role of different B cell subsets, in the periphery and CNS during the lifespan of MS, will be of paramount significance for the understanding of the pathogenesis of the disease*.’

 With reference to the comments to **Table 1**, titled ‘B cell subsets surface markers’, the reviewer is right in his assertion that the motivation for the presentation of this table was the recognition of the significant diversity in the terminology and nomenclature used for the identification of the B cell subtypes in different organs (we used the term ‘compartment’ in the table) described by at least nine different articles cited in our review (references 12, 20, 21, 27, 40, 85,88, 96 and 101). According to the data presented in the table, we also believe that surface markers of B-cells in MS could be specific. Minor changes were introduced to the table including the meaning of the ‘compartment’ heading and a clarification in the ‘compartment’ column for the ‘centroblast’ and ‘centrocyte’ cell markers (the abbreviation ‘GC’ was replaced by ‘CSF’ in both [reference 96]).
